# High-performance double-network ionogels enabled by electrostatic interaction†

**DOI:** 10.1039/c9ra09632a

**Published:** 2020-03-02

**Authors:** Yawen Zhang, Li Chang, Peiru Sun, Ziquan Cao, Yong Chen, Hongliang Liu

**Affiliations:** School of Metallurgy and Materials Engineering, Chongqing University of Science and Technology Chongqing 400050 P. R. China yongchen998@163.com; CAS Key Laboratory of Bio-inspired Materials and Interfacial Science, Technical Institute of Physics and Chemistry, Chinese Academy of Sciences Beijing 100190 P. R. China liuhl@mail.ipc.ac.cn; State Key Laboratory of Applied Organic Chemistry, Key Laboratory of Nonferrous Metal Chemistry and Resources Utilization of Gansu Province, Department of Chemistry, Lanzhou University Lanzhou 730000 P. R. China; Key Laboratory of Bio-Inspired Smart Interfacial Science and Technology of Ministry of Education, School of Chemistry, Beihang University No. 37 Xueyuan Road, Haidian District Beijing 100191 P. R. China

## Abstract

Production of highly conductive and mechanically robust ionogels is urgently needed for the development of diverse flexible electrical devices, but it remains challenging. Herein, we report a facile strategy to prepare high-performance ionogels (ionic conductivity of 1.9 S m^−1^, fracture strain of 170%) *via* electrostatic interaction between mechanically robust charged gel double networks and conductive ionic liquids. Ionogels based on charged polymer networks (with electrostatic interaction) exhibit obvious higher optical transmittance, ionic conductivity, and better mechanical properties compared with those based on neutral polymer networks (without electrostatic interaction). Ionic conductivity and mechanical properties of the ionogels can also be regulated by the double-network structure of the gels. We further develop an ionic skin sensor with the high-performance ionogels used as ionic conductors, which can exhibit excellent sensing performance even under harsh conditions. We envision that this new class of high-performance ionogels would be an attractive alternative to traditional hydrogels, and would extend the applications of ionic conductors to extreme environments.

## Abstract 2

A universal strategy to produce high-performance ionogels has been developed by considering electrostatic interactions between polymer double networks and IL.

## Graphical abstract

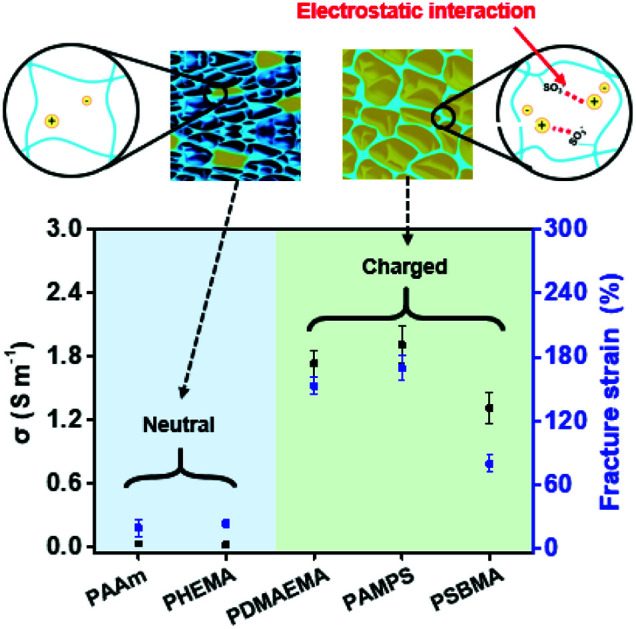

## Introduction

Ionogels^1,2^—a kind of flexible material featuring ionic conductivity and thermal stability, have received an upsurge of interest for potential applications in a wide variety of fields such as field effect transistors,^3–6^ solid electrolytes^7–12^ and dielectric elastomer transducers.^13–15^ When ionogels are used in these electronic devices, high ionic conductivity and good mechanical strength are two key factors that affect their performance. Generally, the existing methods for preparing ionogels including polymerizing ionic liquid (IL) monomers^16–18^ and immobilizing free ILs in polymeric networks or colloidal particles,^19–22^ cannot meet these two criteria simultaneously. To be specific, ionogels fabricated by directly polymerizing IL monomers exhibit relatively low ionic conductivity,^23^ because the transport of ions is greatly restricted after polymerization. In contrast, ionogel prepared by infusing free ILs into polymer networks or colloidal particles can efficiently improve the ionic conductivity, comparable to that of neat ILs. However, these kinds of ionogels suffer drawbacks of weak mechanical strength due to the weak intermolecular interactions between polymer networks and ILs, hindering their applications in flexible electronics.^24^ To overcome these problems, researchers have proposed several approaches to gain mechanical robust ionogels with high conductivity. For example, tough ABA-triblock-based ionogel with high ionic conductivity of 1 S m^−1^ has been fabricated by self-assembly of ABA triblocks in ILs with a subsequent chemically cross-linked annealing step.^25^ Besides, semi-interpenetrating polymer network or organic/inorganic double networks^26^ infused with ILs can also form high-performance ionogels with high mechanical strength and good ionic conductivity. Although great progresses have been made in preparing high-performance ionogels, it is still urgent to develop universal design principles to produce highly conductive ionogels without sacrificing mechanical strength.

Electrostatic interaction, one of the universal weak interactions in nature, plays an essential role in biological processes such as protein–protein interactions,^27^ protein–nucleic acid interactions^28^ and conduction of ions through transmembrane channels and binding of metals or charged ligands.^29^ For example, electrostatic interactions between blocks of oppositely charged residues are the important driving forces for the phase separation of intrinsically disordered regions containing proteins,^30,31^ which forms a miniature reactor in cell to improve the velocity and specificity of biological reaction. Considering that ILs are composed of charged cations and anions, introduction of electrostatic interactions in producing robust ionogels systems might be a powerful tool. On the other hand, double networks,^32–34^ general composed of rigid and brittle first (1^st^) network, and soft and loosely cross-linked second (2^nd^) network, have been demonstrated to be an efficient method to enhance mechanical strength of polymer gels. Recently, we coincidentally found that mechanically robust poly(2-acrylamide-2-methylpropanesulfonic acid) (PAMPS) could effectively hold an IL, *i.e.*, 1-ethyl-3-methylimidazolium dicyanamide ([EMIM][DCA]) to form ionogels both with good mechanical strength and high conductivity.^35^

We proposed that the electrostatic interactions between negatively charged PAMPS networks and [EMIM][DCA] might be responsible to the generated high performance. In this work, to validate the role of electrostatic interaction in the formation of high-performance ionogels, we synthesize a series of ionogels by locating [EMIM][DCA] into charged (positive, negative, or zwitterionic) and neutral polymer double networks, and compare their conductivity and mechanical properties. We find that ionogels based on charged polymer networks (with electrostatic interaction) exhibit obvious higher optical transmittance, ionic conductivity, and better mechanical properties compared with that based on neutral polymer networks (without electrostatic interaction). The ionogels based on charged polymer networks are also stable enough in a wide range of temperatures and can be used as ionic skin under harsh conditions, which may open a new way for special applications in flexible electronics, such as scientific research in very cold or hot regions.

## Experimental section

### Materials

2-Hydroxyethyl methacrylate (HEMA, 99%), acrylamide (AAm, 99%), 2-(dimethylamino)ethyl methacrylate (DMAEMA, 99%), 2-acrylamide-2-methylpropanesulfonic acid (AMPS, 99%) and 3-[dimethyl-[2-(2-methylprop-2-enoyloxy)ethyl]azaniumyl]propane-1-sulfonate (SBMA, 99%) were purchased from Sigma-Aldrich. α-Ketoglutaric acid (KGA 99%), *N*,*N*′-methylenebisacrylamide (NNMBA, 98%) were purchased from J&K Scientific. 1-Ethyl-3-methylimidazolium dicyanamide ([EMIM][DCA]) was purchased from Lanzhou Institute of Chemical Physics (LICP) and have a purity > 99%. Water content in [EMIM][DCA] is determined by using Karl Fischer Moisture Titrator (Mettler Toledo V20, Mettler Toledo Shang Hai Co., Ltd), and the water content is about 0.84 wt%. All the reagents were used without further modification unless specially mentioned.

### Preparation of double-network (DN) hydrogels

DN hydrogels were synthesized by a two-step sequential network formation technique. We use PAMPS-1-4/3-0.01 DN hydrogel as representative to illustrate the preparation process. First, 1 M AMPS containing 4 mol% crosslinking agent NNMBA, and 0.1 mol% initiator α-ketoglutaric acid was confined in a reaction cell which was constructed by two parallel glass plates with 3 mm spacing. Second, the reaction system was exposed to UV for 30 min to yield a single PAMPS-network hydrogel (Fig. S1a†). The UV light (365 nm, 250 W nominal powers) was supplied by Beijing Perfectlight Technology Co., Ltd, and the distance between the sample and the light source was fixed at 40 cm. Then, the single PAMPS-network hydrogel was immersed in an aqueous solution of 3 M AMPS (containing 0.01 mol% NNMBA and 0.1 mol% α-ketoglutaric acid) for 24 h until equilibrium was reached and exposed to UV for 30 min to form the second network. Thus, we realize the fabrication of the DN hydrogel (Fig. S1b†).

### Fabrication of DN ionogel

We prepared the DN ionogels by placing the DN hydrogels in an excess of IL (*i.e.*, [EMIM][DCA]) to allow water-IL exchange, and then keeping at 80 °C under vacuum to ensure complete removal of water. Taking PAMPS-1-4/3-0.01 DN ionogel as an example, firstly, the PAMPS-1-4/3-0.01 DN hydrogel was immersed in deionized water for 24 h (replacing the deionized water every 4 hours), to remove unreacted reactants. The hydrogel was then immersed in pure [EMIM][DCA] to make the substitution of water by [EMIM][DCA] for at least 2 h (replacing IL every 30 min). Subsequently, the ionogel was placed under vacuum at 80 °C for 24 h until the weight of the sample remained unchanged.^35^ DN ionogel film was fabricated after blowing excess [EMIM][DCA] away with nitrogen (Fig. S1c†).

## Results and discussion

We fabricated the ionogels by directly infusing a commercial IL (*i.e.*, 1-ethyl-3-methylimidazolium dicyanamide ([EMIM][DCA])) into charged double polymer networks. The preparation process includes three steps (Fig. S1†). First, the charged polymer double networks were obtained by sequentially polymerizing two different concentrations of monomer-aqueous with *N*,*N*′-methylene-bis-acrylamide and 2,2′-diethoxyacetophenone as the crosslinker and photo initiator respectively. Second, the as-prepared double networks were immersed in pure [EMIM][DCA] to make the substitution of water by [EMIM][DCA]. This substitution process can well proceed for two reasons. On the one hand, the good compatibility between [EMIM][DCA] and water makes this substitution process occur. On the other hand, electrostatic interactions between the charged double polymer networks and [EMIM][DCA] will greatly enhance the speed of this substitution process and keep the [EMIM][DCA]-located polymer networks stable. Third, the generated ionogels were kept at 80 °C under vacuum for 24 h to completely remove the unsubstituted water.

**Fig. 1:**
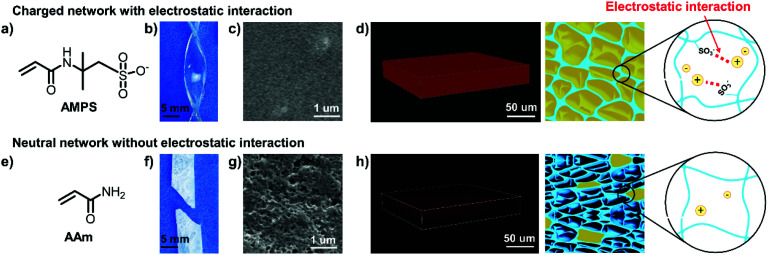
Electrostatic interaction-mediated fabrication of high-performance ionogels. The negative monomer of AMPS (a) generates transparent and flexible ionogel by virtue of the electrostatic interaction between negative PAMPS network and infused [EMIM][DCA] (b). (c) ESEM image of the PAMPS-based DN ionogel is uniform, indicating good compatibility between PAMPS network and [EMIM][DCA]. (d) CLSM image shows that the PAMPS network is full infused with [EMIM][DCA], probably owing to the strong electrostatic interaction between sulfonate groups in the PAMPS double network and imidazolium-based cations. The neutral monomer of AAm (e) generates opaque and fragile ionogel without electrostatic interaction (f). (g) ESEM image of the PAAm-based DN ionogel shows obvious micro pores, which are owing to the presence of water during frozen drying the ionogel for ESEM observation, indicating that water cannot be completely substituted by [EMIM][DCA]. (h) CLSM image of the PAAm-based DN ionogel shows that only a few [EMIM][DCA] are locked in the polymer network.

As a demonstration, we chose negatively charged 2-acrylamido-2-methyl-1-propanesulfonic acid salt (AMPS) (Fig. 1a) as the monomer for both the 1st and 2nd networks to construct DN ionogel. The as-prepared ionogel exhibit high optical transparency with transmittance higher than 90% in the visible light region (Fig. S2†). In addition, the poly(2-acrylamido-2-methyl-1-propanesulfonic acid) (PAMPS)-based DN ionogel also exhibits outstanding mechanical strength, benefiting from the PAMPS double networks. It can be easily crimped without damage (Fig. 1b) and the fraction strain of this ionogel can reach 170%. These characteristics mainly benefit from the good compatibility between the charged PAMPS networks and [EMIM][DCA], which can be determined by the ability of [EMIM][DCA] to swell the PAMPS networks through the substitution of water by [EMIM][DCA]. Fig. 1c shows that the environmental scanning electron microscope (ESEM) image of the PAMPS-based DN ionogel is highly uniform, indicating the PAMPS double network can be swollen with [EMIM][DCA]. To further quantitatively study the ability of [EMIM][DCA] to swell PAMPS double networks, we prepared a series of ionogels with different IL contents by regulating the time of as-prepared PAMPS-based DN hydrogel immersed in pure [EMIM][DCA]. It shows that IL content of PAMPS-based DN ionogel increased with increasing the [EMIM][DCA] substitution time and up to 71% (wt%) IL content can be achieved within 2 h (Fig. S3†). The PAMPS double network that is swollen with [EMIM][DCA] can also be verified by the strong red fluorescence of confocal laser scanning microscopy (CLSM) for PAMPS-based DN ionogel (Fig. 1d), where the [EMIM][DCA] were dyed red with Nile red. We consider the good compatibility between [EMIM][DCA] and PAMPS network may primarily result from the strong electrostatic interactions between the negative charged sulfonate groups of PAMPS double network and the positive charged [EMIM] groups in [EMIM][DCA] (Fig. 1d). Thus, we use neutral acrylamide (AAm) (Fig. 1e) as the monomer to construct DN ionogel with the same method as PAMPS-based DN ionogel (*i.e*., PAAm-based DN ionogel) as control. The PAAm-based DN ionogel is opaque and fragile (Fig. 1f) with porous microstructure (Fig. 1g). Theses micro pores are ascribed to the presence of water during frozen drying the ionogel for ESEM observation, indicating that most water cannot be substituted by [EMIM][DCA] in the PAAm double networks. CLSM further shows that only a few [EMIM][DCA] are locked in the polymer network (Fig. 1h). These results demonstrate the importance of electrostatic interaction in constructing transparent and flexible ionogel. The strong electrostatic interaction also leads to high IL content for PAMPS-based DN ionogel, which further results in high ionic conductivity. The ionic conductivity of the PAMPS-based ionogel is as high as 1.9 S m^−1^, far greater than that of PAAm-based ionogel (0.03 S m^−1^). Moreover, our PAMPS-based DN ionogel exhibits higher ionic conductivity (1.9 S m^−1^) and better fraction strain (170%) than previous ionogels based on EMIMCl/HEMA/CS/water^36^ (0.2 S m^−1^, 100%) and EMIMTFSI/PEGMA/PEGDA^37^ (0.24 S m^−1^, 40%) system. Recently, Kim *et al.*^38^ reported an ionic mechanoreceptor skin which composed of hydrogen-bonded [EMIM][TFSI] ion pairs on the surface of silica microstructures embedded into thermoplastic polyurethane elastomeric matrix, exhibiting a higher fraction strain of 800%, but the ionic conductivity of the ionogel (0.007 S m^−1^) is much lower than that of our ionogel. Yan *et al.*^39^ further reported an ionogel with high ultimate tensile strain of 1390% and ionic conductivity of about 0.8 S m^−1^ at 25 °C by a simple method using thiol–ene click chemistry under mild condition. Overall, our PAMPS-based DN ionogel exhibits outstanding ionic conductivity while maintaining relatively good mechanical strength. More importantly, the PAMPS-based DN ionogel can keep excellent ionic conductivity (1.2–5.3 S m^−1^) in a wide temperature range from −80 to 80 °C (Fig. S4a†). The ionic conductivity keeps relatively stable in the temperature ranging from −40 to 40 °C. A sharp increase of ionic conductivity occurs with further increasing temperature from 40 to 80 °C, which is probably owing to the decreased viscosity of [EMIM][DCA]^40^ and thus easier transport of ions at high temperatures. It should be noted that the ionogel still keeps good conductivity (1.2 S m^−1^) even at −80 °C, which is lower than the crystallization temperature of [EMIM][DCA].^41^ This phenomenon may benefit from the strong electrostatic interactions between PAMPS double network and [EMIM][DCA], which shield the crystallization of [EMIM][DCA]. Moreover, the ionogel can keep long-term stability with ionic conductivity scarcely changed even for 28 d (Fig. S4b†).

**Fig. 2:**
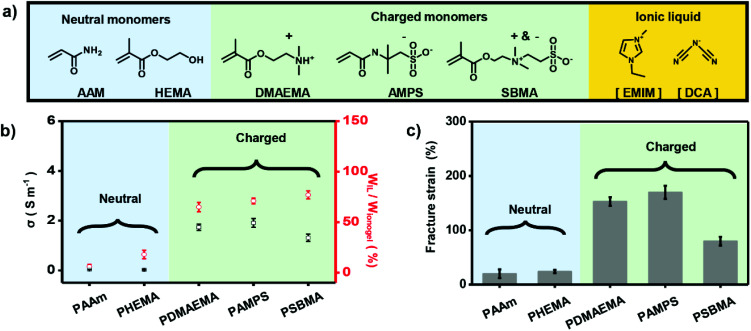
Electrostatic interaction-enhanced high-performance ionogels. (a) Chemical structures of neutral monomers, charged monomers and IL used in this work. (b) Charged polymer (CP)-based DN ionogels exhibit obvious higher IL content and ionic conductivity than neutral polymer (NP)-based DN ionogels. (c) Fracture strains of the CP-based DN ionogels are far greater than that of the NP-based DN ionogels.

To further demonstrate the importance of electrostatic interaction between polymer networks and IL in making high-performance ionogels, we used a series of monomers including neutral AAm, 2-hydroxyethyl methacrylate (HEMA), positively charged 2-(dimethylamino) ethyl methacrylate (DMAEMA), negatively charged AMPS, and zwitterionic 3-[dimethyl-[2-(2-methylprop-2-enoyloxy)ethyl]azaniumyl]propane-1-sulfonate (SBMA) (Fig. 2a) to fabricate different ionogels. As shown in Fig. 2b, ionogels based on charged polymer double networks (*i.e.*, PDMAEMA, PAMPS, and PSBMA) (CP-based DN ionogels) exhibit obvious higher IL content compared with ionogels based on the neutral polymer networks (*i.e.*, PAAm and PHEMA) (NP-based DN ionogels), which can also be verified by the strong and uniform red fluorescence of CLSM for the CP-based DN ionogels (Fig. S5†), and weak and nonuniform fluorescence of CLSM for NP-based DN ionogels (Fig. S6†). These results indicate the electrostatic interaction between polymer networks and IL can greatly enhance the capacity of polymer networks to adsorb IL. The higher IL content for CP-based DN ionogels compared with NP-based DN ionogels further results in higher ionic conductivity. We used PAMPS-based DN ionogels as an example to systematically investigate the relationship between IL content and ionic conductivity. The IL contents of PAMPS-based DN ionogel can be well controlled by regulating the [EMIM][DCA] substitution time. It shows that ionic conductivity of PAMPS-based DN ionogels increases with increasing the IL content (Fig. S7†). In addition to IL content, ionic conductivity of CP-based DN ionogels is also influenced by the capability of free ion mobility, which can be regulated by the charge type of polymer networks. As shown in Fig. 2b, although IL contents of all CP-based ionogels are about 70%, ionic conductivity of negatively, positively charged and zwitterionic polymer-based DN ionogel ((NCP, PCP and ZP)-based DN ionogel) is different. For PCP-based DN ionogel, the ionic conductivity is dominated by the [EMIM] cations because the [DCA] anions are restricted by the PCP network through strong electrostatic interactions. In contrast, the ionic conductivity of NCP-based DN ionogels relies more on [DCA] anions owing to restricted mobility of [EMIM] cations. Larger [EMIM] cations (volume of 182 Å^3^)^42^ are expected to be drifting more slowly than smaller [DCA] anions (volume of 86 Å^3^),^42^ thus leading to slightly lower ionic conductivity of PCP-based DN ionogel (PDMAEMA ionogel) than NCP-based DN ionogel (PAMPS ionogel). In the case of the ZP-based DN ionogel, although its slight larger IL content, it exhibits the lowest ionic conductivity because the mobility of both [EMIM] cations and [DCA] anions is restricted in some extent. The strong electrostatic interaction also facilitates the stable location of IL in the polymer networks, which further results in strong mechanical properties. In this work, we used dumbbell-shaped sample to test the fraction strains (Fig. S8†). As shown in Fig. 2c, fraction strains of CP-based DN ionogels are in the range of 80–170%, far greater than that of the NP-based DN ionogels (20–24%). Therefore, we can conclude that the electrostatic interaction between charged polymer networks and IL is critical important to form ionogels with higher ionic conductivity and better mechanical strength.

**Fig. 3:**
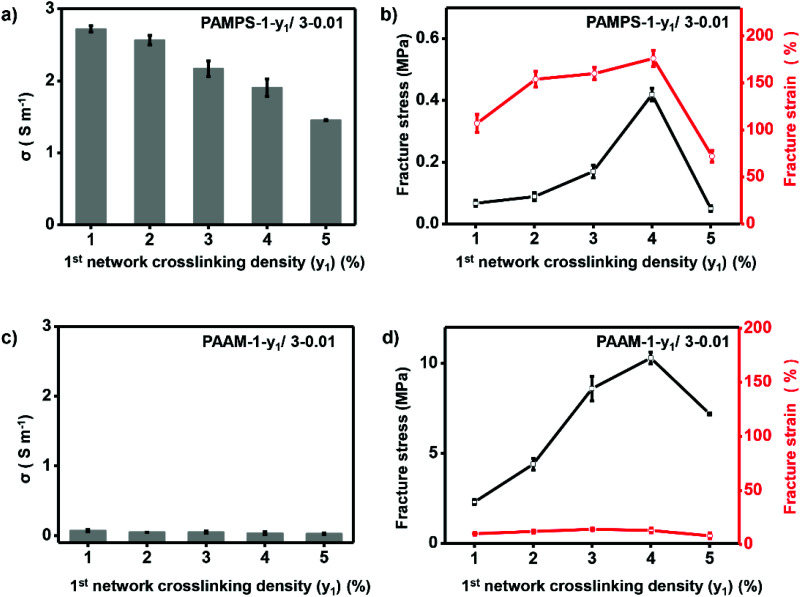
Optimizing crosslinking density of the 1^st^ network. For charged PAMPS-based DN ionogel, increasing 1^st^ network crosslinking density leads to slight decrement of the ionic conductivity (a), and maximum fracture stress and strain are achieved with 4% crosslinking density of the 1^st^ network (b). For neutral PAAm-based DN ionogel, ionic conductivity (c) and fracture strain (d) always stay in low values with varying 1^st^ network crosslinking density.

Ionic conductivity and mechanical properties are among the most important parameters for high-performance ionogels, and were thus optimized in detail. In this work, we found these parameters can be easily optimized by adjusting the double network structure including the 1^st^ network crosslinking density and molar ratio of the 2^nd^ to the 1^st^ network. The double-network ionogels are referred to as P-*x*_1_-*y*_1_/*x*_2_-*y*_2_, where P, *x*_*i*_ and *y*_*i*_ (*i* = 1, 2 for the 1^st^ and 2^nd^ network respectively) are the abbreviated polymer name, molar monomer concentration and crosslinking density (molar ratio of crosslinker/monomer (%) for *i*-th network) respectively. We fixed the molar monomer concentration of the 1^st^ network with 1, and molar monomer concentration and crosslinking density of the 2^nd^ network with 3 and 0.01% respectively (P-1-*y*_1_/3-0.01) to investigate the effect of the 1^st^ network crosslinking density (*y*_1_) on ionic conductivity and mechanical strength of the ionogels. As shown in Fig. 3a, increasing *y*_1_ of the charged PAMPS-1-*y*_1_/3-0.01 from 1% to 5% leads to decrease of the ionic conductivity from 2.7 to 1.4 S m^−1^, which is probably owing to the lower IL content in the polymer matrix with increasing crosslinking density. The IL content of the ionogel decreased from 76% to 53% with increasing crosslinking density from 1% to 5% (Table S1†). Different from the ionic conductivity, mechanical properties (*i.e.*, fracture stress and fracture strain) of this kind of ionogels increase first and then decrease, and reach maximum values when the 1^st^ network crosslinking density is 4% (Fig. 3b). To balance mechanical properties and ionic conductivity with the desire for practical applications, we chose *y*_1_ of 4% as the optimal conditions to achieve ionic conductivity of 1.91 S m^−1^, fracture stress of 0.0419 MPa, and fracture strain of 174%. In contrast to the charged PAMPS-1-*y*_1_/3-0.01 with adjustable ionic conductivity and mechanical properties, ionogels based on neutral polymer networks cannot achieve appropriate ionic conductivity and mechanical properties by adjusting *y*_1_. As shown in Fig. 3c and d, ionic conductivity and fracture strain of PAAm-1-*y*_1_/3-0.01 keep very low value (0.03–0.07 S m^−1^, 8–14%) with increasing *y*_1_ from 1% to 5%.

**Fig. 4:**
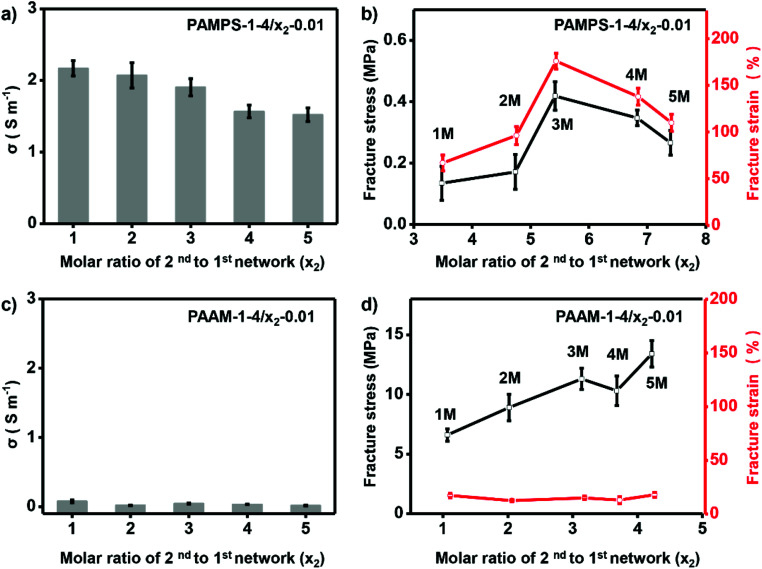
Optimizing molar ratio of 2^nd^ to 1^st^ network. For charged PAMPS-based DN ionogel, increasing molar ratio of 2^nd^ to 1^st^ network leads to slight decrease of the ionic conductivity (a), and maximum fracture stress and strain are achieved when the molar ratio of 2^nd^ to 1^st^ network is 5.42 (b). For neutral PAAm-based DN ionogel, ionic conductivity (c) and fracture strain (d) keep very low values independent on molar ratio of 2^nd^ to 1^st^ network. Numbers on the curves denote the value of 2^nd^ network monomers concentrations, *x*_2_.

To further investigate the effect of molar ratio of 2^nd^ to 1^st^ network on performance of the ionogels, we prepared ionogels of P-1-4/*x*_2_-0.01. The different molar ratios of 2^nd^ to 1^st^ networks were obtained by immersing the 1st network gel into solutions of 2^nd^ network monomers with various concentrations (*x*_2_). As shown in Fig. 4a, increasing molar ratio of 2^nd^ to 1^st^ network of PAMPS-1-4/*x*_2_-0.01 (increasing *x*_2_) results in decrease of the ionic conductivity (from 2.17 to 1.52 S m^−1^). However, fracture strain and stress of PAMPS-1-4/*x*_2_-0.01 increase first and then decrease with *x*_2_ = 3 as optimum (Fig. 4b). It should be noted that only ionogels prepared with charged polymer networks can be adjusted with molar ratio of 2^nd^ to 1^st^ network. The fracture strain and ionic conductivity of NP-based DN ionogel (PAAm-1-4/*x*_2_-0.01) keep very low value during the adjustment of molar ratio of 2^nd^ to 1^st^ network (Fig. 4c and d). As the high ionic conductivity and good mechanical properties are important to the final application, we thus choose *y*_1_ = 4% and *x*_2_ = 3 to prepare the charged PAMPS-based DN ionogel (PAMPS-1-4/3-0.01) for further study.

**Fig. 5:**
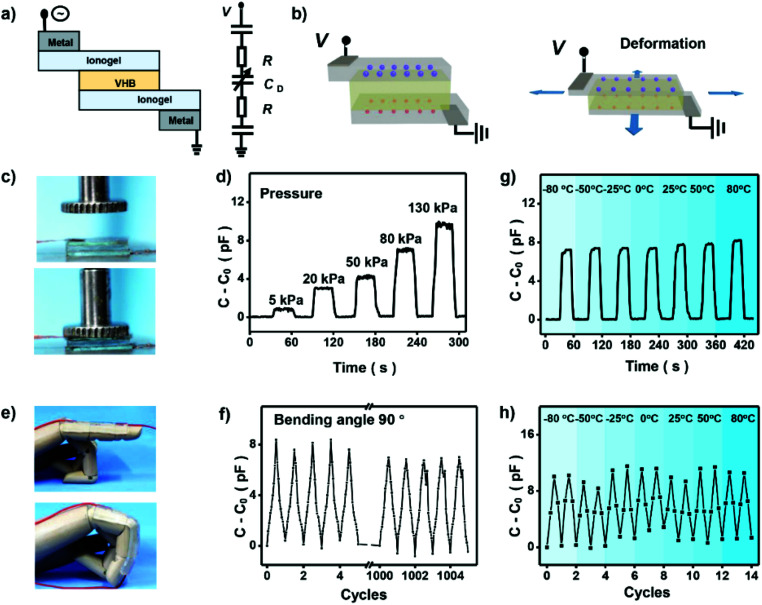
Ionic skin sensor based on PAMPS-based DN ionogel. (a) Basic design of the ionic skin, which is a dielectric of VHB sandwiched by two layers of ionogels. Two metals are connected to outside of the deformable area of the ionogel as electrodes. (b) The capacitance changed with external forces deforming the dielectric when a small voltage is applied to the capacitance. (c) The ionic skin was pressed with a precise pressure. (d) Capacitance of the ionic skin increase with increasing the pressure. (e) The ionic skin was assembled to a straight finger of mechanical hand and bended from 0° to 90°. (f) Capacitance of the ionic skin responds stably with bending from 0° to 90° and can be repeated more than 1000 times. Capacitance of the ionic skin responds stably to stress (g) and strain (h) in a wide temperature range from −80 °C to 80 °C.

To demonstrate the potential application of the high-performance ionogels in elastic electronic devices, capacitance based ionic skin sensor was prepared. Fig. 5a is a schematic illustrating the layers of the finished ionic skin sensor. The ionic skin sensor was prepared by using ionogel as the ionic conductor, and an acrylic elastomer (VHB 4905, 3M) as the dielectric. The PAMPS-1-4/3-0.01 ionogel shows adequate adhesion to the finger and can be easily stretched by bending the finger. In contrast to common ionic skin sensors based on hydrogels that need two other layers to prevent the evaporation of water from the hydrogels,^43^ the ionogel is stable enough and only three layers are needed for the final ionic skin sensor. When applied a voltage on the sensor, an electrical double layer at the interface between two ionic conductors forms, which behaves like a capacitor. When external forces to press or bend the dielectric, the capacitance of this circuit changed (Fig. 5b), which enables the device to sense the deformation. As shown in Fig. 5c and d, we press the ionic skin with a precise pressure and record the capacitance of the ionic skin with a simple portable capacitance meter. It shows that capacitance of the ionic skin increases linearly with increasing pressure (Fig. S9†). This linear relationship endows us to detect the pressure directly using measured capacitance. We also assembled this ionic skin to a straight finger of a mechanical hand (Fig. 5e). As the finger bends, deformation of the ionic skin occurs to adapt the motion at the joints. This deformation is large enough for daily finger movement, and performance of the ionic skin remains its origin state with almost no obvious damage by repeating this kind of large deformation. We recorded the capacitance of the ionic skin with repeatedly bending the finger from 0° to 90°. Fig. 5f shows that capacitance of the ionic skin responds stably to mechanical motion and can be repeated more than 1000 times. Significantly, our ionic skin also exhibits outstanding sensing performance under harsh conditions. For example, the ionic skin as stress and strain sensors can be performed well in wide temperatures ranging from −80 °C to 80 °C (Fig. 5g and h). This unique property of the ionic skin may solve the problem that hydrogel-based ionic skin sensors will lose their function at lower or higher temperatures. Thus, our designed ionogel will broaden the application of ionic skin in special areas, such as scientific investigation in very cold or hot regions.

## Conclusions

In conclusion, we have developed a new kind of high-performance ionogels with excellent transparency, good mechanical strength, and high conductivity enabled by electrostatic interaction-mediated IL locking in charged double polymer networks. By rational regulating the chemical compositions of polymer networks for ionogels, we conclude that electrostatic interaction is a key factor to enhance the performance of the ionogels. The designed ionogels can also tolerate a wide range of temperatures and can be further used as ionic skin under harsh conditions. This study provides a new idea to fabricate high-performance soft materials for flexible electronics, especially under harsh conditions.

## Conflicts of interest

There are no conflicts to declare.

## Supplementary Material

RA-010-C9RA09632A-s001
