# Supplementary material for: High-performance double-network ionogels enabled by electrostatic interaction
Source: RSC Adv. 2020 Mar 2;10(13):7424–31. doi: 10.1039/c9ra09632a (PMC9049851; doi:10.1039/c9ra09632a)
Supplement: RA-010-C9RA09632A-s001 [file RA-010-C9RA09632A-s001.pdf]

Electronic Supplementary Material (ESI) for RSC Advances.
This journal is © The Royal Society of Chemistry 2020

High-Performance Double-Network Ionogels Enabled by Electrostatic Interaction

Yawen Zhang,[‡a,b] Li Chang,[‡b,c] Peiru Sun,[‡a,b] Ziquan Cao,[d] Yong Chen,[*,a] and Hongliang Liu[*,b]

[a]School of Metallurgy and Materials Engineering, Chongqing University of Science and Technology.

[b]CAS Key Laboratory of Bio-inspired Materials and Interfacial Science, Technical Institute of Physics and Chemistry, Chinese Academy of Sciences.

[c]State Key Laboratory of Applied Organic Chemistry, Key Laboratory of Nonferrous Metal Chemistry and Resources Utilization of Gansu Province and Department of Chemistry, Lanzhou University.

[d]Key Laboratory of Bio-Inspired Smart Interfacial Science and Technology of Ministry of Education, School of Chemistry, Beihang University.

[‡]These authors contribute equally.

1. Preparation of double-network (DN) ionogels

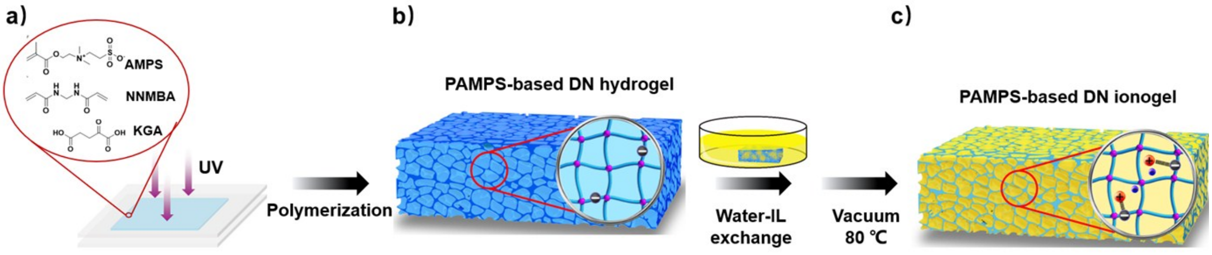

Figure S1. Fabrication processes of the PAMPS-based DN ionogel. The PAMPS-based DN ionogel was obtained through a three-step method, including constructing PAMPS-based hydrogel, exchanging water with IL and subsequent vacuum treatment at 80 °C.

2. Determining molar ratio of the second network to the first network

We obtained molar ratio of the second network to the first network by the following method. First, we achieved the mass fraction of the polymer network (η_1) in a single-network hydrogel as follow:

$$\eta_1 = \frac{m_{sf}}{m_{sh}} \times 100\% \tag{1}$$

where m_{sf} is the weight of the dried framework, which was obtained by freeze drying the single network hydrogel, and m_{sh} is the weight of the single network hydrogel before drying. Second, DN hydrogel was prepared by using a newly-fabricated single-network hydrogel (weight of $m_{sh'}$) with the same composition as the aforementioned single-network hydrogel. In this case, the weight of the first dried framework can be calculated as $m_{sh'} \times \eta_1$. The as-prepared DN hydrogel was freeze dried and the weight of the dried framework (m_{df}) was obtained. The weight of the second dried framework should be m_{df} - $m_{sh'} \times \eta_1$. So, molar ratio of second network to first network can be expressed as:

$$x = \frac{m_{df} - m_{sdsh'} \times \eta_1}{m_{sh'} \times \eta_1} \tag{2}$$

3. Determining IL content of the ionogels

IL content of the DN ionogels (C_{IL}) can be expressed as mass ratio of swollen IL to the DN ionogel and can be expressed as follow:

$$C_{IL} = \frac{m_i - m_d \times \eta_2}{m_i} \times 100\% \tag{3}$$

where m_i is the weight of the DN ionogel, m_d is the weight of the DN hydrogel, and η_2 is the framework mass ratio of the DN hydrogel. To obtain η_2, we prepared a new DN hydrogel with weight of m_{d1} and dried framework weight of m_{df1} by freeze drying. The framework mass ratio of the DN hydrogel was calculated as follow

$$\eta_2 = \frac{m_{df1}}{m_{d1}} \times 100\% \tag{4}$$

Table S1. Molar ratio of the 2nd to 1st network and IL content of PAMPS-based ionogels

Second network molar monomer concentration, x_2 (mol L^{-1})	Molar ratio of the 2nd to 1st network	IL content (C_{IL})
1	3.48	76%
2	4.74	69%
3	5.42	65%
4	6.82	58%
5	7.39	53%

4. Ionic conductivity and optical transmittance measurements

Ionic conductivity of the ionogels was measured using a four-probe resistance measuring instrument (RTS-9, Guangzhou Four-probe Tech Co., LTD). The four probes of the four-probe resistance meter are linearly distributed with equal spacing (s = 1 mm) and a

probe radius of 500 μm. The outer pair of probes are current carrying electrodes and the inner pair of probes are voltage sensing electrodes. The visible light transmittance of the ionogels was measured using a UV-vis spectrophotometer (UV-2600, SHIMADZU Co., LTD,) at 400 nm to 800 nm. As show in figure S2, the transmittance of the PAMPS-based DN ionogel can reach more than 90% in the visible light region.

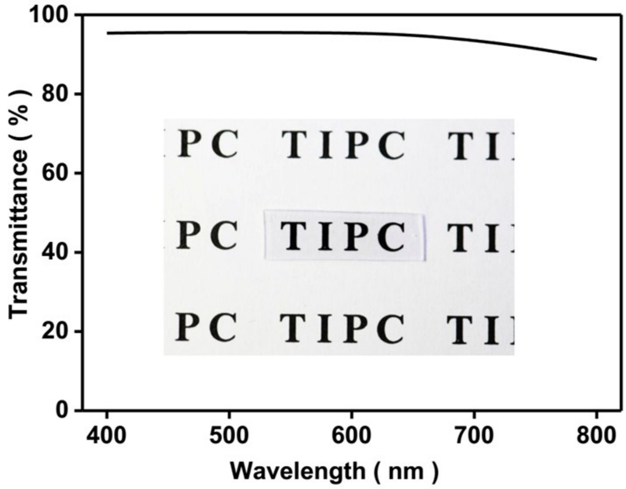

Figure S2. Transmittance of the PAMPS-based DN ionogel can reach more than 90% in the visible light region.

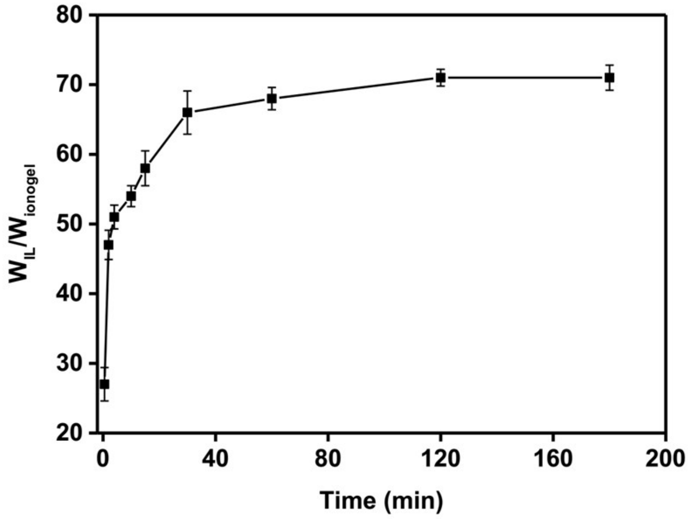

Figure S3. IL content of PAMPS-based DN ionogel increased with increasing the [EMIM][DCA] substitution time and up to 71% (wt%) IL content can be achieved within [EMIM][DCA] substitution time of 120 min.

5. Conductivity stability of the PAMPS-based DN ionogel

We tested conductivity of the PAMPS-based DN ionogel in the temperature from -80 °C to 80 °C. As show in Figure S4a, the PAMPS-based DN ionogel can keep excellent

ionic conductivity (1.2-5.3 S m^{-1}) in a wide temperatures range from −80 to 80 °C. We also tested the conductivity change of PAMPS-based DN ionogel under ambient conditions for 28 days. As show in Figure S4b, our ionogel can keep long-term stability with ionic conductivity scarcely changed even for 28 days.

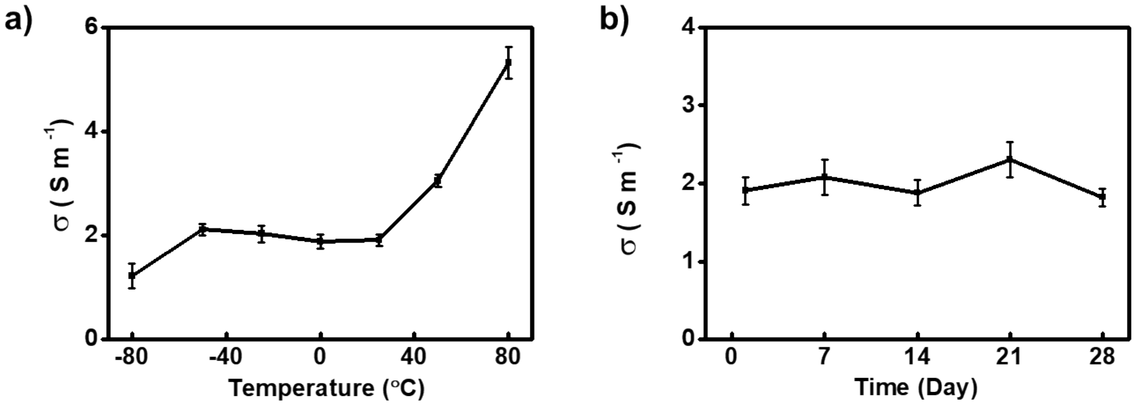

Figure S4. (a) The PAMPS-based DN ionogel shows relatively high ionic conductivity over a wide temperature range from −80 to 80 °C. (b) Ionic conductivity of the ionogel is scarcely changed even for 28 d, indicating good long-term stability.

6. Morphologies of the ionogels

The morphologies of the ionogels were characterized by using a confocal laser scanning microscopy (CLSM). The CLSM was carried out using a Nikon laser confocal-structured illumination super-resolution fluorescence microscope (N-C2-SIM, Nikon Instruments Co., LTD). We placed the DN hydrogel in deionized water containing methyl blue for 2 h to dye the polymer network blue. Then, the hydrogel was placed in [EMIM][DCA] (dyed with Nile red) to achieve complete substitution of water by dyed [EMIM][DCA] to obtain a test sample. CLSM shows strong and uniform red fluorescence for the CP-based DN ionogels (Figure S5), and weak and nonuniform fluorescence for NP-based DN ionogels (Figure S6). The morphologies of the ionogels were also characterized using environmental scanning electron microscopy (ESEM) (QUANTA FEG 250, FET Co., TED). The ESEM measurement was performed with an accelerating voltage of 5 KV.

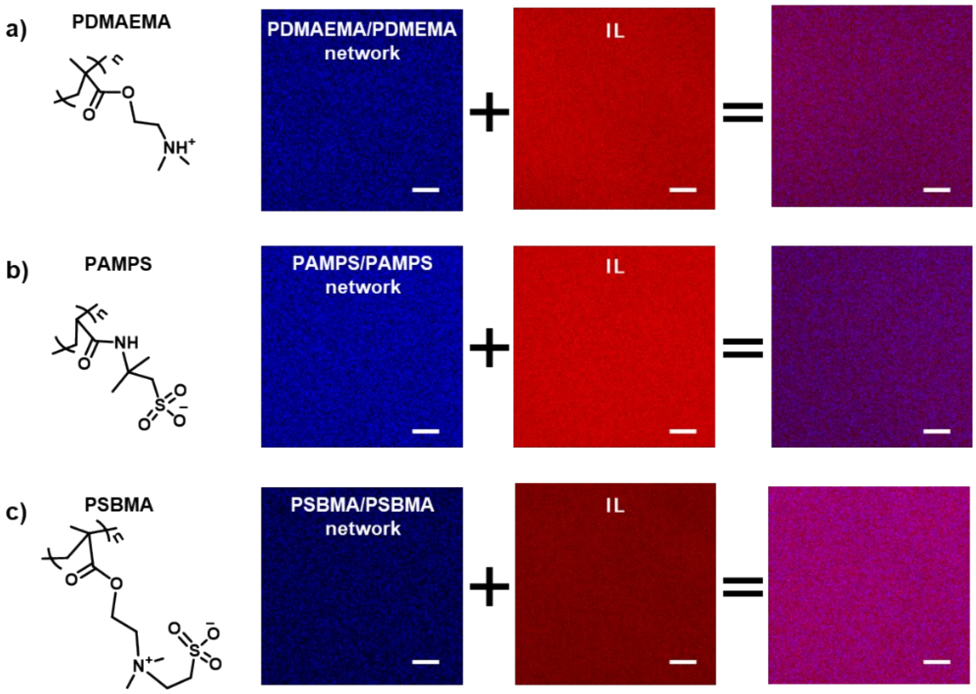

Figure S5. CLSM images of the CP-based DN ionogels with PDMAEMA (a), PAMPS (b) and PSBMA (c). The red fluorescence for [EMIM][DCA] is strong and uniform. Scale bar: 20 μm.

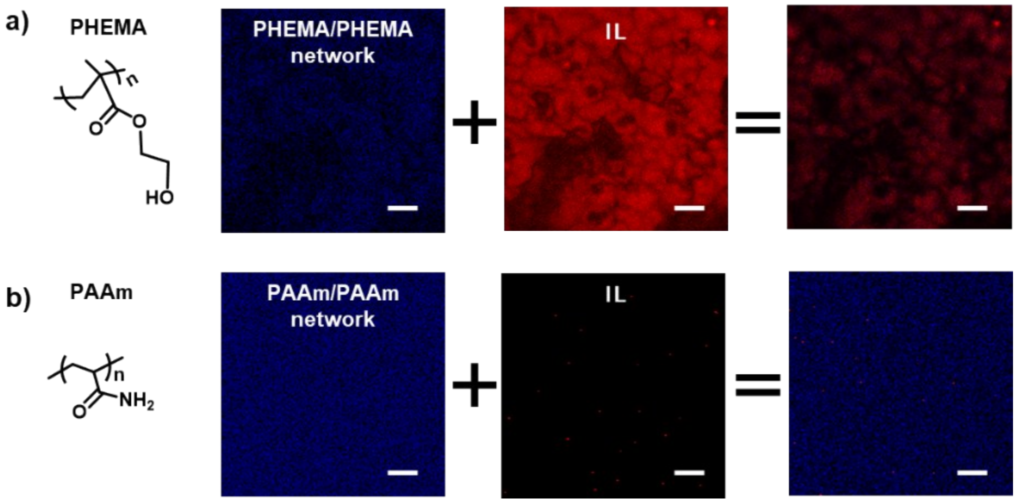

Figure S6. (a) CLSM images of the NP-based DN ionogels with PHEMA (a), PAAm (b). The red fluorescence for [EMIM][DCA] is nonuniform or weak. Scale bar: 20 μm.

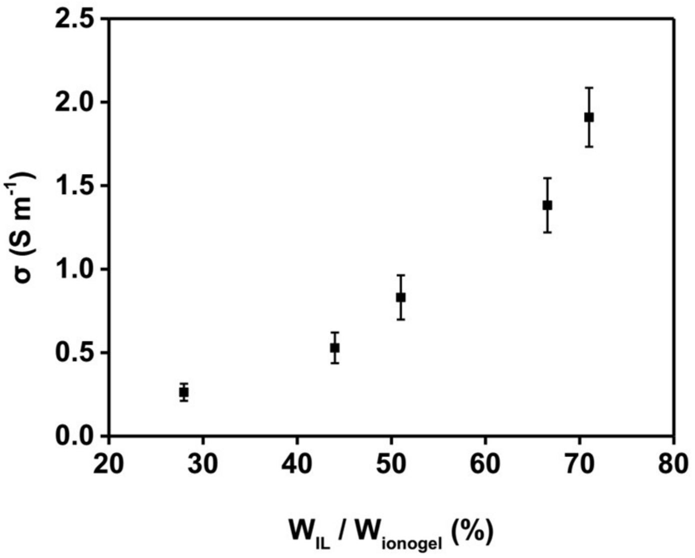

Figure S7. Ionic conductivity of PAMPS-based DN ionogels increases with increasing the IL content

7. Tensile Measurements:

Tensile measurements of the ionogels were performed on a tensile-compressive tester (Tensilon ESM303, Beijing Jeepin Times Technology Co., LTD,). In the tensile test, each testing sample was cut into dumbbell shape (Figure S8). The thickness depends on the sample itself, and stretching speed is 5 mm/min.

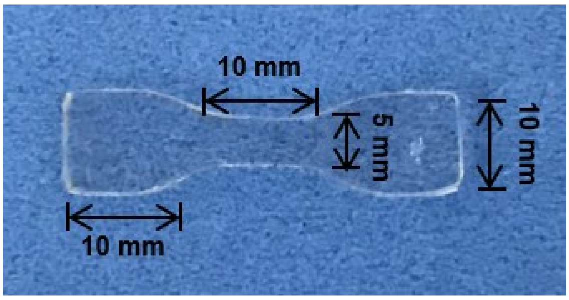

Figure S8. Image of the dumbbell-shaped testing sample.

S9).

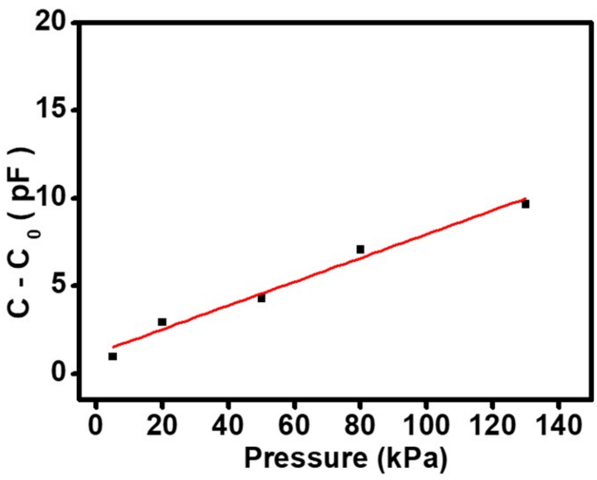

Figure S9. The capacitance changes of the ionic skin increase linearly with increasing pressure.

8. Applying the PAMPS-based DN ionogel to a sensor

We prepared the PAMPS-based DN ionogel into a capacitive sensor, and attached the sensor to an artificial joint to monitor mechanical motion in the temperature range of −80 to 80 °C. A handheld portable capacitance meter (MT 4800D, Suzhou Dong Wei Yuan Electronic Co., LTD) was connected to the sensor trough two long electric wires to test the capacitance changes during movements. For measurements at low temperatures from -80 °C to 0 °C, the ionogel sensor was frozen in a cryogenic refrigerator (MDF-U33868, Anhui Zhong Ke Du Ling Commercial Electrical Appliances LTD) for 30 min. Then we put the cyclic bending artificial joint in the cryogenic refrigerator and attach the sensor to it. For the measurement at a high temperature range from 30 °C to 80 °C, the designed ionogel was heated in an oven (DZF 6050, Shang Hai Yi Heng Scientific Instrument Co., LTD) for 30 minutes. Then we placed the cyclic bending artificial joint in the oven and connected the sensor to handheld portable capacitance meter. When we press the ionic skin with a precise pressure and record the capacitance of the ionic skin with the simple portable capacitance meter, capacitance of the ionic skin increases linearly with increasing pressure (Figure S9).
